# Socioeconomic characteristics and relapse-free and overall survival from childhood cancer – a nationwide study based on data from the Danish Childhood Cancer Registry

**DOI:** 10.2340/1651-226X.2025.42131

**Published:** 2025-01-29

**Authors:** Fie Stegenborg, Mathilde S. Bek, Charlotte J. Nilsson, Line H. Pedersen, Thomas Scheike, Lisa L. Hjalgrim, Friederike Erdmann, Kjeld Schmiegelow, Pernille Bidstrup, Line Kenborg, Jeanette F. Winther, Hanne B. Larsen, Susanne O. Dalton

**Affiliations:** a Cancer Survivorship, Danish Cancer Institute, Copenhagen, Denmark; b Section of Social Medicine, Department of Public Health, University of Copenhagen, Copenhagen, Denmark; c Section of Biostatistics, Department of Public Health, University of Copenhagen, Copenhagen, Denmark; d Department of Pediatrics and Adolescent Medicine, Copenhagen University Hospital, Rigshospitalet, Copenhagen, Denmark; e Research Group Etiology and Inequalities in Childhood Cancer, Division of Childhood Cancer Epidemiology, Institute of Medical Biostatistics, Epidemiology and Informatics (IMBEI), University Medical Center of the Johannes Gutenberg University Mainz, Mainz, Germany; f Department of Prevention and Evaluation, Leibniz Institute for Prevention Research and Epidemiology – BIPS, Bremen, Germany; g Department of Clinical Medicine, Faculty of Medicine, University of Copenhagen, Copenhagen, Denmark; h Psychological Aspects of Cancer, Cancer Survivorship, Danish Cancer Institute, Copenhagen, Denmark; i Department of Psychology, University of Copenhagen, Copenhagen, Denmark; j Childhood Cancer Research Group, Danish Cancer Institute, Copenhagen, Denmark; k Department of Clinical Medicine, Faculty of Health, Aarhus University and Aarhus University Hospital, Aarhus, Denmark; l Department of Clinical Oncology & Palliative Care, Zealand University Hospital, Naestved, Denmark

**Keywords:** Childhood cancer, socioeconomic position, social inequality, relapse, cancer survival, register-based study, socioeconomic status

## Abstract

**Background and purpose:**

Over the past decades, childhood cancer survival has increased substantially in Europe, including Denmark. However, families with fewer social resources may have benefitted less from these improvements. In this nationwide register-based study, we assessed associations between parental socioeconomic position (SEP) and 5-year relapse-free survival (RFS) and overall survival (OS) in childhood cancer patients.

**Material and methods:**

All children aged <16 years diagnosed with cancer in Denmark between 1998 and 2017 were identified in the Danish Childhood Cancer Registry (N = 3245). Parents, with whom the children resided, were identified, and data on the parents’ education, cohabitation status, affiliation to work market, country of origin, and vital status of the children were obtained through individual-level linkage across Danish nationwide registries. Cox proportional hazards models were used to estimate the association between SEP indicators and 5-year RFS and OS.

**Results and interpretation:**

Tendencies towards lower 5-year RFS and OS were observed among children whose parents were unemployed/not in workforce (RFS: HR [hazard ratio] = 1.14, 95% CI [confidence interval]: 0.90–1.45, OS: HR = 1.28, 95% CI: 0.95–1.71) or from non-Western countries (RFS: HR = 1.21 95% CI: 0.96–1.52, OS: HR = 1.44, 95% CI: 1.09–1.90). Results by diagnostic groups revealed particularly low OS for children with non-central nervous system tumors whose parents were from non-Western countries (HR = 1.92, 95% CI: 1.24–2.97). Targeted strategies are needed to promote social equity and ensure optimal diagnosis, care, and management of childhood cancer across social groups.

## Introduction

Survival from childhood cancer has improved considerably over the past decades and exceeds 80% in most of Europe [1] but childhood cancer still remains the leading cause of disease-related deaths in children aged 1–15 years in Europe [2]. Previous studies suggest that not all children benefit equally from the advancements in diagnostics and treatment of childhood cancer, and socioeconomic disparities in survival from childhood cancer have been observed across countries, including in European countries [3–10], where children in principle should have equal access to healthcare. Specifically, lower survival has been observed in children with cancer whose parents have a low income [4, 5] or short education [4, 6, 7] and in children with only one caregiver in the household [9]. Similar to social inequality observed in overall survival after childhood cancer, poorer event-free survival has been observed in children with acute lymphocytic leukemia (ALL) from families with low socioeconomic position (SEP) compared to high SEP [11]. However, the literature regarding social inequalities and relapse of childhood cancer is scarce. Since the 1990s, standardized guidelines for the treatment of childhood cancer have been introduced in the Nordic countries [12], whereas, in case of relapse of childhood cancer, children are still treated heterogeneously to a high degree [13]. Even though relapse-free survival is used for evaluating the progression of cancer disease [14], no previous study has, to our knowledge, comprehensively examined the association between socioeconomic inequalities and relapse-free survival for all childhood cancers combined. Moreover, the development in diagnostics, treatment, supportive care options, and ultimately survival warrants updated knowledge on socioeconomic disparities in childhood cancer survival, including characterization of potentially vulnerable families.

In this nationwide register-based study, we add to the limited evidence about social inequalities in relapse-free and overall survival after childhood cancers. We examine the associations between numerous measures of parental SEP and relapse-free and overall 5-year survival, respectively, in Danish children diagnosed with cancer from 1998 to 2017.

## Material and methods

### Data sources

We identified all incident childhood cancers in the Danish Childhood Cancer Registry, which holds information on practically all diagnosed cancers in children living in Denmark since 1985 [15]. The unique personal identification number (PIN) assigned to all Danish residents since 1968 allowed us to link to individual level information across Danish administrative and health registries [16]. Using the children’s PIN, the children were linked to their respective household addresses and the parent(s) they were living with at the date of diagnosis. The children were linked to the parent(s) registered at the same address to reflect the social resources of the household rather than the SEP of the biological parents. Information from social registries was obtained to assess different parental SEP indicators at the household level. SEP is determined by various factors that may affect an individual’s social and economic status within a given society, such as education, income, or occupation [17]. We used the highest attained education of the parent in the household and categorized it as short (<10 years; primary and lower secondary education), medium (10–12 years; upper secondary and vocational), and long education (>12 years; higher education); parental affiliation to the work market was categorized as working, unemployed/not in workforce (if parents were either unemployed or outside the work market entirely (i.e. unable to work)), and mixed (if parents belonged to different categories); parental cohabitation status was categorized as living together (married or cohabiting) or living alone; parental country of origin was categorized as Denmark, non-Western countries, and Western countries or mixed (if parents belonged to different categories). To ensure temporality, information regarding parental education and parental affiliation to the work market was obtained 1 year before diagnosis, while information on parental cohabitation status was obtained at the year of diagnosis to reflect the parents’ living arrangements throughout the disease management of the child. Information on emigration and vital status was obtained from the Danish Civil Registration System [18], information on relapse was obtained from the Danish Childhood Cancer Registry [15], and information on cause of death was obtained from the Danish Register of Causes of Death [19].

### Study population

The study population comprised all children (aged 0–15 years) diagnosed with a first primary cancer between 1st of January 1998 and 31st of December 2017. In total, 3448 children were registered with a childhood cancer diagnosis. We excluded 154 children who were diagnosed with Langerhans Cell Histiocytosis, due to uncertainties in the definition of the disease [20]. Moreover, we excluded 27 children who had no registered parent(s) at their address at the date of diagnosis. Of the remaining children, we excluded 22 children with missing data on parental SEP indicators or if they had a registered relapse prior to the date of diagnosis, as this was likely a registry error. Thus, we included 3245 children for analysis (Supplementary Figure 1). Based on the 12 diagnostic main groups of the International Classification of Childhood Cancer (ICCC) 3rd edition [21], we defined the following three diagnostic groups to be used in our analysis: Hematological malignancies (leukemia and lymphomas; ICCC groups 1 and 2), CNS tumors (ICCC group 3), and non-CNS solid tumors (ICCC groups 4–12) (Supplementary Table 1).

### Statistical analysis

The outcome in the relapse-free survival analysis was the first (if any) relapse or death within 5 years after diagnosis. The outcome in the overall survival analysis was death from any cause within 5 years after diagnosis. Children were followed from date of diagnosis until date of relapse (only in the relapse-free survival analyses), death, date of emigration, date of 5 years of follow-up, or end of study (31st of December 2019), whichever came first.

Kaplan–Meier curves and *p*-values of log-rank tests were used to compare differences in relapse-free and overall survival probabilities between groups of SEP indicators. Cox proportional hazards models were used to estimate the association between SEP and survival. We performed overall 5-year survival analyses by diagnostic groups. Additionally, we estimated Kaplan–Meier curves to visually explore trends in survival over time by parental country of origin, both for all cancers combined and by diagnostic groups.

We performed separate analyses for overall 5-year survival stratified by child’s age at diagnosis and sex. We estimated cumulative incidence functions and performed cause-specific hazards models with up to 22 years follow-up to estimate cause-specific death by SEP indicators. Individuals with no information on cause of death were excluded from the analysis (*n* = 5). Thus, the study population in the cause-specific survival analysis consisted of 3240 individuals. Cause of death was categorized into death as a result of cancer and death as a result of other causes (comprising non-cancer natural deaths, suicide, and other non-natural deaths) based on International Classification of Diseases (ICD) codes version 8 and 10 (Supplementary Table 2).

Analyses of the association between highest parental education, parental affiliation to work market, and parental cohabitation status, respectively, and survival were adjusted for year of diagnosis, age of mother at diagnosis, and parental country of origin. Analysis of the association between parental country of origin and survival was adjusted for year of diagnosis, age of mother at diagnosis, and highest parental education (Supplementary Figure 2). If there was no registered mother in the household, we adjusted for age of father at diagnosis. Results were expressed as hazard ratios (HRs) with 95% confidence intervals (CIs).

The proportional hazards assumption for all models was examined using Schoenfeld residuals test [22]. Moreover, we tested for linearity between the log hazard and each continuous covariate using Martingale residual plots [22]. In some of the models, the assumptions of proportional hazards and linearity were not met for the continuous covariate of mother’s age at diagnosis. We categorized mother’s age at diagnosis into four categories (≤25 years, 26–29 years, 30–39 years, and ≥40 years) and performed separate analyses. The HRs only changed marginally, and we thus used mother’s age at diagnosis as a continuous variable. Moreover, we observed non-linearity for year of diagnosis. Thus, we likewise categorized year of diagnosis into four categories (1998–2002, 2003–2007, 2008–2012, and 2013–2017) and performed separate analyses. However, the HRs of the indicators of SEP and the outcome of interest only changed marginally, and therefore we used year of diagnosis as a continuous variable. All statistical analyses were performed using the statistical software R, version 4.1.2 (R Core Team, 2020) [23].

### Results

Of the 3245 children with cancer included in the present study, 45% were diagnosed before the age of 5 years, and 53% were males. Moreover, 5% had a mother who was younger than 25 years of age at the child’s diagnosis, 10% lived in households with short parental education, 15% with single parents, and 10% lived with parents who were unemployed/not in the workforce. Most of the children lived with parents whose country of origin was Denmark (85%), while 10% lived with parents who were both from non-Western countries (Table 1).

**Table 1:** Characteristics of children diagnosed with cancer in 1998–2017 in Denmark by main diagnostic groups and for all childhood cancers combined.

	Hematological malignancies (*N* = 1288)	CNS tumors (*N* = 848)	Non-CNS solid tumors (*N* = 1109)	Total (*N* = 3245)
**Age at diagnosis**				
0–4	592 (46%)	306 (36%)	576 (52%)	1474 (45%)
5–10	395 (31%)	304 (36%)	244 (22%)	943 (29%)
11–15	301 (23%)	238 (28%)	289 (26%)	828 (26%)
**Sex**				
Female	511 (43%)	415 (49%)	549 (49%)	1515 (47%)
Male	737 (57%)	433 (51%)	560 (51%)	1730 (53%)
**Period of diagnosis**				
1998–2002	315 (24%)	234 (28%)	293 (26%)	842 (26%)
2003–2007	346 (27%)	183 (22%)	274 (25%)	803 (25%)
2008–2012	325 (25%)	219 (26%)	274 (25%)	818 (25%)
2013–2017	302 (23%)	212 (25%)	268 (24%)	782 (24%)
**Age of mother at time of diagnosis (years)**				
≤25	44 (3%)	31 (4%)	75 (7%)	150 (5%)
26–30	170 (13%)	104 (12%)	199 (18%)	473 (15%)
31–40	730 (57%)	471 (55%)	562 (51%)	1763 (54%)
≥41	322 (25%)	228 (27%)	258 (23%)	808 (25%)
Household without mother	22 (2%)	14 (2%)	15 (1%)	51 (2%)
**Highest parental education**				
Long	595 (46%)	385 (45%)	513 (46%)	1493 (46%)
Medium	541 (42%)	372 (44%)	468 (42%)	1381 (43%)
Short	152 (12%)	91 (11%)	128 (12%)	371 (11%)
**Parental cohabitation status**				
Living as a couple	1094 (85%)	712 (84%)	950 (86%)	1756 (85%)
Living alone	194 (15%)	136 (16%)	159 (14%)	489 (15%)
**Parental affiliation to work market**				
Working	951 (74%)	644 (76%)	798 (72%)	2393 (74%)
Unemployed/not in workforce	127 (10%)	80 (9%)	106 (10%)	313 (10%)
Mixed	210 (16%)	124 (15%)	205 (19%)	539 (17%)
**Parental country of origin**				
Denmark	1089 (85%)	725 (86%)	932 (84%)	2746 (85%)
Western countries or mixed countries	74 (6%)	46 (5%)	66 (6%)	186 (6%)
Non-Western countries	125 (10%)	77 (9%)	111 (10%)	313 (10%)

**Figure 1:**
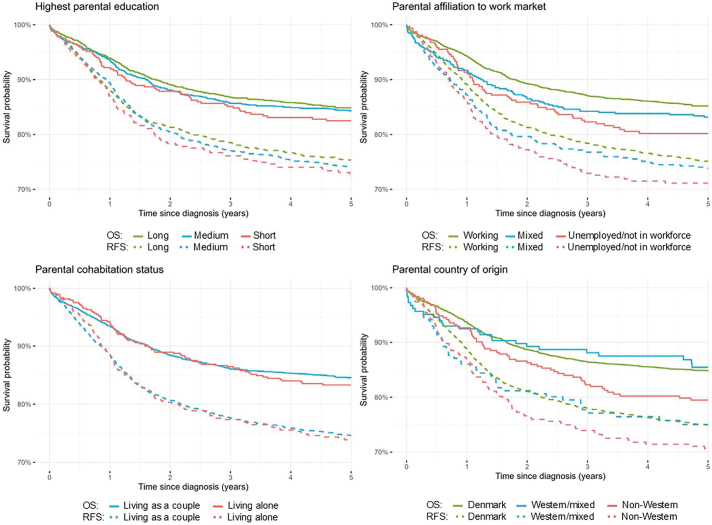
Kaplan–Meier curves for relapse-free 5-year survival (RFS) and overall 5-year survival (OS) in children with childhood cancer by measures of socioeconomic position (SEP).

In the 5-year relapse-free survival analysis the study population was followed for a total of 12,612 person-years, and in the 5-year overall survival analysis the study population was followed for a total of 13,771 person-years (Table 2). Approximately 1 year after diagnosis, survival probabilities diverged by parental education with the lowest relapse-free survival probability (log-rank = 0.6) and lowest overall survival probability (log-rank = 0.5) observed among children with short parental education. The pattern was confirmed by results of the Cox proportional hazards models, but upon adjustment no associations were seen. Regarding parental affiliation to the work market, the relapse-free survival probability (log-rank = 0.2) and overall survival probability (log-rank = 0.03) were lowest for children with unemployed parents/parents not in workforce (Figure 1). The same tendency was seen in the Cox proportional hazards models with statistically significantly lower overall survival in children whose parents were unemployed/not in the workforce (unadjusted HR = 1.38, 95% CI: 1.05–1.81). Upon adjustment, the HR did not reach statistical significance; however, the estimate was still considerably elevated (Table 2). No associations were observed for relapse-free survival and overall survival by parental cohabitation status. Among the groups of country of origin, children with parents from non-Western countries had the lowest relapse-free survival probability (log-rank = 0.2) and lowest overall survival probability (log-rank = 0.06). A higher HR of relapse and death (adjusted HR = 1.21, 95% CI: 0.96–1.52) and a higher HR of death including all causes (adjusted HR = 1.44, 95% CI: 1.09–1.90) was seen in children with parents from non-Western countries compared to children with parents from Denmark (Figure 1 and Table 2).

**Table 2:** Measures of SEP and relapse-free 5-year survival and overall 5-year survival in children with childhood cancer.

	Relapse-free survival^a^						Overall survival^b^					
			Unadjusted		Adjusted				Unadjusted		Adjusted	
	
	Person years	Events (*N*)	HR	95% CI^c^	HR^d,e^	95% CI^c^	Person years	Events (*N*)	HR	95% CI^c^	HR^d,e^	95% CI^c^
**Highest parental education**												
Long	5769	360	1.00	Ref.	1.00	Ref.	6303	221	1.00	Ref.	1.00	Ref.
Medium	5428	353	1.05	0.91, 1.22	1.02	0.88, 1.19	5905	215	1.05	0.87, 1.27	0.96	0.79, 1.17
Short	1415	99	1.12	0.90, 1.40	1.04	0.82, 1.31	1563	64	1.18	0.89, 1.55	1.03	0.77, 1.38
**Parental affiliation to work market**												
Working	9396	584	1.00	Ref.	1.00	Ref.	10,254	349	1.00	Ref.	1.00	Ref.
Unemployed/not in work force	1156	89	1.21	0.97, 1.52	1.14	0.90, 1.45	1276	61	1.38	1.05, 1.81	1.28	0.95, 1.71
Mixed	2060	139	1.08	0.90, 1.30	1.04	0.86, 1.26	2241	90	1.17	0.93, 1.48	1.15	0.90, 1.46
**Parental cohabitation status**												
Living as a couple	10,726	687	1.00	Ref.	1.00	Ref.	11,701	420	1.00	Ref.	1.00	Ref.
Living alone	1887	125	1.03	0.85, 1.24	1.03	0.85, 1.24	2070	80	1.07	0.84, 1.36	1.08	0.85, 1.37
**Parental country of origin**												
Denmark	1769	676	1.00	Ref.	1.00	Ref.	11,715	411	1.00	Ref.	1.00	Ref.
Western countries or mixed countries	686	45	1.02	0.75, 1.37	1.03	0.76, 1.40	772	26	0.95	0.64, 1.41	1.04	0.70, 1.55
Non-Western countries	1157	91	1.23	0.98, 1.53	1.21	0.96, 1.52	1283	63	1.38	1.05, 1.79	1.44	1.09, 1.90

SEP: socioeconomic position; HR: hazard ratios; CI: confidence intervals.

a Event was defined as relapse or death from all causes.

b Event was defined as death from all causes.

c Corresponding 95% confidence intervals.

d Highest parental education, parental affiliation to work market and parental cohabitation status were adjusted for year of diagnosis, age of mother at time of diagnosis, and parental country of origin.

e Parental country of origin was adjusted for year of diagnosis, age of mother at time of diagnosis, and highest parental education.

In overall survival analyses by diagnostic groups, we found tendencies toward higher HRs of death from hematological malignancies in children with short parental education than high parental education and in children whose parents had mixed affiliation to the work market or were unemployed/not in workforce than children with working parents. Similar tendencies toward higher HRs of death were observed in children with CNS tumors whose parents were unemployed/not in the workforce compared to those who were working or were from non-Western countries than from Denmark. In addition, we found statistically significantly higher HRs of death in children diagnosed with non-CNS solid tumors with parents from non-Western countries than parents from Denmark (adjusted HR = 1.92, 95% CI: 1.24–2.97) (Table 3). When exploring survival trends by diagnostic period, we observed less of an increase in survival for children with parents from non-Western countries compared to those with parents from Denmark or mixed countries in recent periods (Supplementary Figure 3). This pattern was evident for non-CNS solid tumors and CNS tumors but not for hematological malignancies (data not shown due to small numbers). When stratifying by age group, we observed especially elevated HRs of death in children aged 11–15 years whose parents had short education compared to long parental education and in children aged 0–4 years and 11–15 years with parents from non-Western countries compared to Denmark. No modifications were seen according to sex on the 5-year overall survival (Supplementary Tables 3 and 4).

**Table 3:** Measures of SEP and overall 5-year survival by diagnostic group.

	Hematological malignancies^a^						CNS tumors^a^						Non-CNS solid tumors^a^					

			Unadjusted		Adjusted				Unadjusted		Adjusted				Unadjusted		Adjusted	

	Person years	Events (*N*)	HR	95% CI^b^	HR^c,d^	95% CI^b^	Person years	Events (*N*)	HR	95% CI^b^	HR^c,d^	95% CI^b^	Person years	Events (*N*)	HR	95% CI^b^	HR^c,d^	95% CI^b^
**Highest parental education**																		
Long	2575	68	1.00	Ref.	1.00	Ref.	1521	81	1.00	Ref.	1.00	Ref.	2206	72	1.00	Ref.	1.00	Ref.
Medium	2385	56	0.91	0.64, 1.29	0.82	0.57, 1.17	1508	79	1.01	0. 74, 1.38	0.93	0.67, 1.27	2012	80	1.22	0.89, 1.68	1.13	0.82, 1.57
Short	646	26	1.53	0.97, 2.40	1.32	0.82, 2.13	376	17	0.88	0.52, 1.48	0.77	0.45, 1.33	541	21	1.19	0.73, 1.93	1.01	0.60, 1.71
**Parental affiliation to work market**																		
Working	4177	100	1.00	Ref.	1	Ref.	2620	127	1.00	Ref.	1.00	Ref.	8258	140	1.00	Ref.	1.00	Ref.
Unemployed/not in work force	540	20	1.51	0.93, 2.44	1.49	0.89, 2.50	298	22	1.47	0.94, 2.32	1.41	0.86, 2.30	1055	21	1.18	0.75, 1.87	1.02	0.62, 1.67
Mixed	889	30	1.40	0.93, 2.10	1.38	0.90, 2.11	488	28	1.19	0.79, 1.78	1.15	0.76, 1.75	2036	37	1.05	0.73, 1.51	1.06	0.73, 1.54
**Parental cohabitation status**																		
Living as a couple	4761	126	1.00	Ref.	1.00	Ref.	2863	148	1.00	Ref.	1.00	Ref.	9788	167	1.00	Ref.	1.00	Ref.
Living alone	846	24	1.06	0.69, 1.65	1.10	0.71, 1.70	542	29	1.03	0.69, 1.53	1.04	0.70, 1.56	1561	31	1.12	0.76, 1.64	1.06	0.72, 1.56
**Parental country of origin**																		
Denmark	4757	127	1.00	Ref.	1.00	Ref.	2928	147	1.00	Ref.	1.00	Ref.	4031	137	1.00	Ref.	1.00	Ref.
Western countries or mixed countries	309	7	0.82	0.38, 1.76	0.89	0.41, 1.91	180	10	1.11	0.58, 2.10	1.19	0.62, 2.26	283	9	0.94	0.48, 1.84	1.03	0.52, 2.03
Non-Western countries	540	16	1.10	0.65, 1.85	1.05	0.61, 1.80	297	20	1.30	0.82, 2.08	1.44	0.89, 2.33	446	27	1.75	1.16, 2.64	1.92	1.24, 2.97

SEP: socioeconomic position; HR: hazard ratios; CI: confidence intervals.

a Event of overall survival was defined as death from all causes.

b Corresponding 95% confidence intervals.

c Highest parental education, parental affiliation to work market and parental cohabitation status were adjusted for year of diagnosis, age of mother at time of diagnosis, and parental country of origin.

d Parental country of origin was adjusted for year of diagnosis, age of mother at time of diagnosis, and highest parental education.

In sub-analyses with 22 years of follow-up, there was an increased cumulative incidence of death due to cancer in children whose parents were unemployed/not in the workforce and were from non-Western countries (Supplementary Figure 4). In line with the results from the relapse-free and overall survival analyses, higher HRs for death due to cancer were observed for children with parents who were unemployed/not in the workforce (adjusted HR = 1.20, 95% CI: 0.88–1.65) and children with parents from non-Western countries than Denmark (adjusted HR = 1.37, 95% CI: 1.02–1.84). Highest effect estimate for death as a result of causes other than cancer was observed among children whose parents had short education (adjusted HR = 1.94. 95% CI: 1.02–3.71) (Supplementary Table 5).

## Discussion

In this nationwide population-based study using high-quality registry data, children diagnosed with cancer whose parents were unemployed/not part of the workforce compared to working and children with parents from non-Western countries compared to Denmark had lower overall 5-year survival, and further tended to have lower relapse-free survival after 5 years.

Although Denmark provides free and equal access to health care services [24], our findings suggest that some socioeconomic inequalities in relapse and survival from childhood cancer exist, most pronounced in children whose parents are unemployed/not in workforce or from non-Western countries. In line with our findings, previous studies from European countries have reported social inequality in overall survival from childhood cancer [3–10], suggesting that children from less advantaged families have lower survival than children from more advantaged families. The literature on socioeconomic inequalities and relapse in children with cancer is scarce. A population-based study carried out in the United States found that the cumulative incidence of relapse in children with ALL did not differ between children living in high- and low-poverty areas. However, the study reported a higher risk of early relapse in children diagnosed with ALL who lived in high-poverty areas than among children who lived in low-poverty areas [25], thus indicating social inequalities in relapse after primary treatment.

### Delayed diagnosis

Evidence from Denmark has demonstrated that children with parents with low compared to high income had more consultations in general practice before the child was diagnosed with cancer [26]. In line with this, another Danish study reported that children with parents with mixed affiliation to the work market and parents of non-Western origin have more frequent use of emergency healthcare contacts prior to their child getting a cancer diagnosis than working parents and parents from Denmark [27]. These findings may suggest that physicians struggle to identify cancer in children from families with fewer resources, potentially leading to delayed diagnosis, more advanced stage at diagnosis, and consequently poorer survival outcomes, although the study did not find any associations between parental SEP and more advanced stage at diagnosis [27]. It may be even more challenging to identify children with cancer in families from foreign countries due to language barriers. Findings indicate that both healthcare professionals and parents of children with leukemia face significant concerns when communicating across languages, leading to major challenges in the clinical setting [28]. Specifically, we observed higher hazard of cancer-related deaths among children whose parents were from non-Western countries than from Denmark in the cause-specific survival analysis. Challenges related to being an immigrant in Denmark may be even more pronounced in more complicated treatment regimes, such as non-CNS solid tumors, which is a heterogeneous group of tumors that often require intensive multidisciplinary therapy tailored to the specific diagnosis [29]. Our explorative analyses suggest that prognostic improvements over time have not equally benefited children with parents from non-Western countries, particularly for those with solid tumors, compared to children with parents from Denmark. Taken together, this might explain the notably lower survival we observed in children with non-CNS solid tumors, whose parents were from non-Western countries compared to those from Denmark.

Moreover, it has been reported that immigrants, particularly those with non-Western backgrounds, may experience difficulties in terms of education and employment [30]. Indeed, we observed an increased risk of relapse and lower survival in children with parents who were unemployed/not in the workforce. Unemployment concerns may further add pressure to the already demanding role of caring for a child with cancer. A family’s social network and resources are vital in the management of a child with cancer [31], and these may be more limited for families with no affiliation to the work market.

### Treatment variation

Although no clear association was observed between parental education and survival in our study for all cancers combined, there was a tendency toward lower survival in children with hematological malignancies and those aged 10–15 years whose parents had lesser compared to higher education. In addition, a study comprising Danish and Swedish children with cancer reported that low maternal education was associated with early mortality (death within <90 days after diagnosis) but not associated with later mortality (death within 1–5 years after diagnosis) [4]. Parental education might influence the family’s ability to demand and negotiate with physicians, potentially leading to differences in childhood cancer outcomes [31]. Differences in parental adherence to medication and treatment recommendations have also been suggested to contribute to the observed disparities, especially in cancers such as ALL, where outpatient maintenance therapy usually lasts several years [31]. However, a Danish study found that while adherence to therapy among families of children diagnosed with ALL did not vary by parental SEP, children whose parents had lesser education or were unemployed received lower doses of maintenance therapy than children whose parents had higher education and were working [32]. This discrepancy may reflect inadequate compliance to protocol recommendations on drug dosage by physicians rather than the family’s adherence to therapy [32].

### Lack of standardized relapse treatment

The observed disparities in survival by parental SEP may further be influenced by the increased complexity of therapies required if a child experiences a relapse. When first-line therapies fail, second-line options are often less established. The lack of standardized treatments can lead to more discussions about the management of the child’s disease, and as a result, the decision to pursue curative treatment may be influenced by the family’s resources and the physicians’ attitudes. Socioeconomic status can play a crucial role in this context, affecting the family’s ability to advocate for and participate in decisions regarding initiating such therapies [31].

### Strengths and limitations of this study

A significant strength of this study is the utilization of data from nationwide registries with nearly complete inclusion of all Danish children with cancer and minimal loss to follow-up. Utilizing data from the Danish Childhood Cancer Registry allowed us to evaluate relapse-free survival and cause-specific death as well as overall survival where only the latter is used as outcome in previous studies. Estimating relapse-free survival, in addition to overall survival, provides a comprehensive understanding of how parental socioeconomic factors influence not only survival after childhood cancer but also the risk of cancer relapse, offering critical insights into how socioeconomic disparities affect childhood cancer prognosis. Exploring cause-specific death allowed us to determine if observed patterns were attributable to cancer-related deaths or influenced by mortality from other causes, noting that even when using deaths from other causes than cancer as competing risk the increased hazard of cancer-related deaths remained evident. Through Danish population-based registries with high validity, we obtained comprehensive information on parental SEP with almost complete coverage, not influenced by self-reports, which resulted in low risk of information bias. Another strength is that we chose to link the children to the parent(s) registered at the same address to reflect the social resources of the household rather than the SEP of the biological parents. Moreover, parental education and parental affiliation to the work market were measured 1 year before the date of the child’s diagnosis, and parental cohabitation status was measured on the year of diagnosis, thus ensuring a temporal causal pathway. It is a strength that we used Cox proportional hazards models and cause-specific hazards models, as we were able to account for censoring [33]. We argue that the external validity of this study is high, especially in other countries with welfare systems similar to the Danish.

Even though we conducted a nationwide population-based study covering 20 years of childhood cancer diagnoses, the cohort is relatively small, and the low number of relapse and death limits statistical power, especially for sub-analyses by diagnostic group. However, although not statistically significant upon adjustment, clear patterns were observed across SEP indicators indicating that this probably is a power issue. Another limitation is that some of the SEP indicators may change during the 5-year follow-up. Nonetheless, we argue that parental SEP at the time of diagnosis most accurately reflects the social resources of the family during diagnostic workup and treatment.

## Conclusion

In this nationwide population-based study with high-quality data from Danish registries, we found that children diagnosed with cancer whose parents were unemployed/not in workforce or were from non-Western countries had lower overall 5-year survival, and further tended to have lower relapse-free survival after 5 years. The mechanisms contributing to these socioeconomic disparities may include delayed diagnosis and differences in treatment driven by how physicians respond to parents’ negotiation skills, as well as insufficient or lacking protocols for treatment of relapse. Targeted strategies are required to promote greater social equity and ensure the best possible diagnosis, care, and management of childhood cancer across social groups.

## Supplementary Material

Socioeconomic characteristics and relapse-free and overall survival from childhood cancer – a nationwide study based on data from the Danish Childhood Cancer Registry

## Data Availability

The data utilized in this study were accessed remotely on a secure platform at Statistics Denmark. Any access to data requires permission from Statistics Denmark and the Danish Cancer Institute. Contact Person: Professor Susanne Oksbjerg Dalton. Email: sanne@cancer.dk.
