# Supplementary material for: Socioeconomic characteristics and relapse-free and overall survival from childhood cancer – a nationwide study based on data from the Danish Childhood Cancer Registry
Source: Acta Oncol. 2025 Jan 29;64:42131. doi: 10.2340/1651-226X.2025.42131 (PMC11808813; doi:10.2340/1651-226X.2025.42131)
Supplement: Socioeconomic characteristics and relapse-free and overall survival from childhood cancer – a nationwide study based on data from the Danish Childhood Cancer Registry [file AO-64-42131-s1.pdf]

Supplementary material has been published as submitted. It has not been copyedited, or typeset by Acta Oncologica

Supplementary

Figure 1: Flowchart of the nationwide cohort of children diagnosed with childhood cancer from 1998-2017 included in the analyses 2

Figure 2: Overview of associations, black arrows indicating the associations of interest 3

Figure 3: Kaplan-Meier curves for overall 5-year survival in children with childhood cancer by parental country of origin, stratified by diagnostic period 4

Figure 4: Cumulative incidence functions for cause of death in children with childhood cancer by indicators of SEP 5

Table 1: Categorization of childhood cancer types based on the ICCC 3rd version diagnostic groups 6

Table 2: Classification of cause of death 7

Table 3: Overall 5-year survival in children with childhood cancer by age groups 8

Table 4: Overall 5-year survival in children with childhood cancer by sex 9

STROBE Statement — Checklist of items that should be included in reports of cohort studies ... 11

Figure 1: Flowchart of the nationwide cohort of children diagnosed with childhood cancer from 1998-2017 included in the analyses

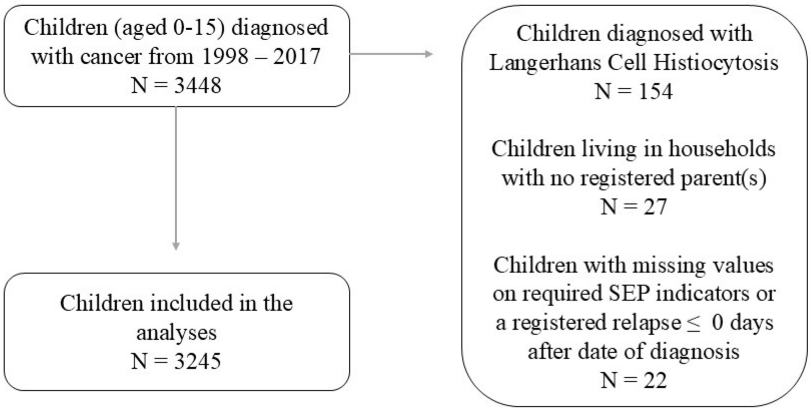

Figure 2: Overview of associations, black arrows indicating the associations of interest

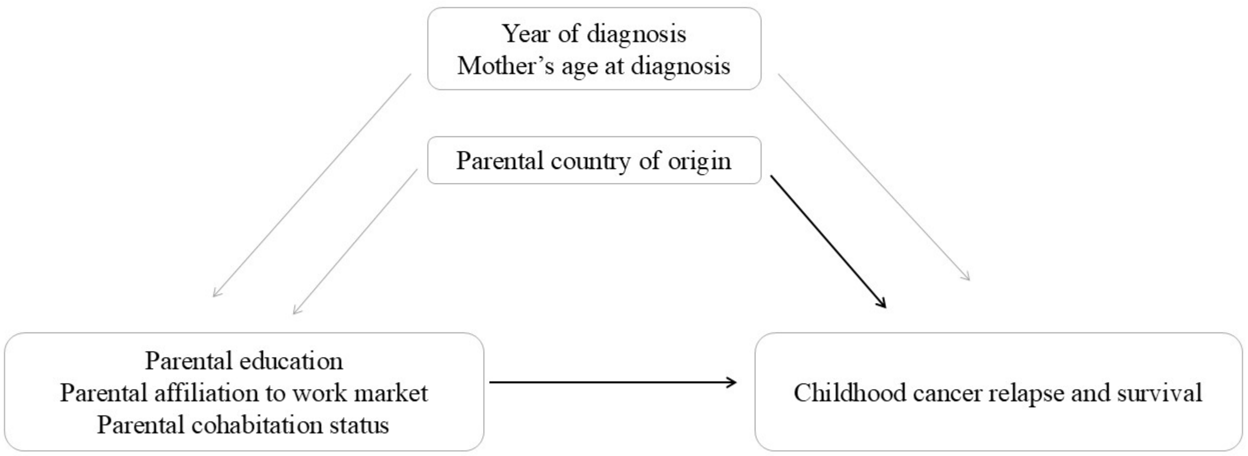

Figure 3: Kaplan-Meier curves for overall 5-year survival in children with childhood cancer by parental country of origin, stratified by diagnostic period

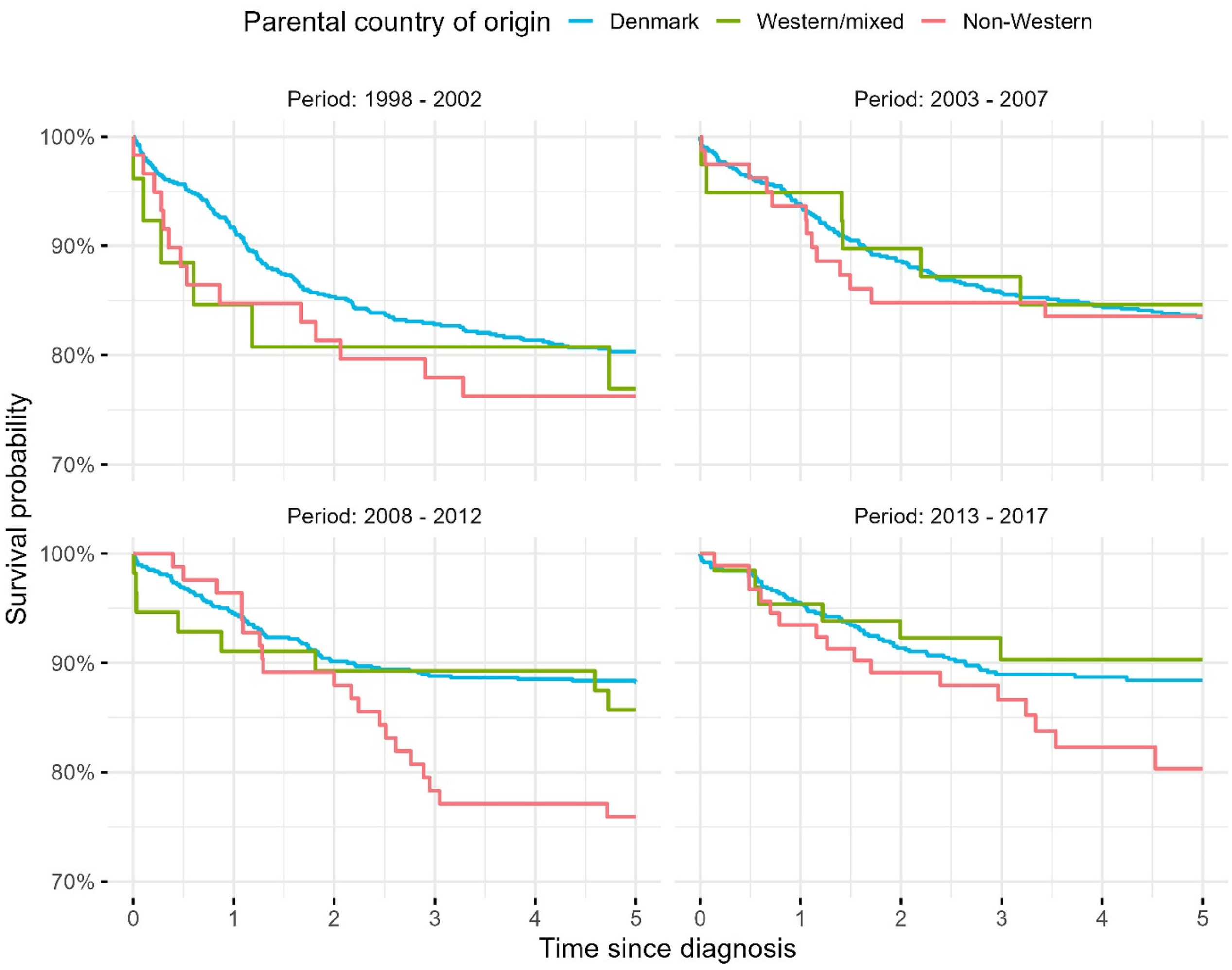

Figure 4: Cumulative incidence functions for cause of death in children with childhood cancer by indicators of SEP

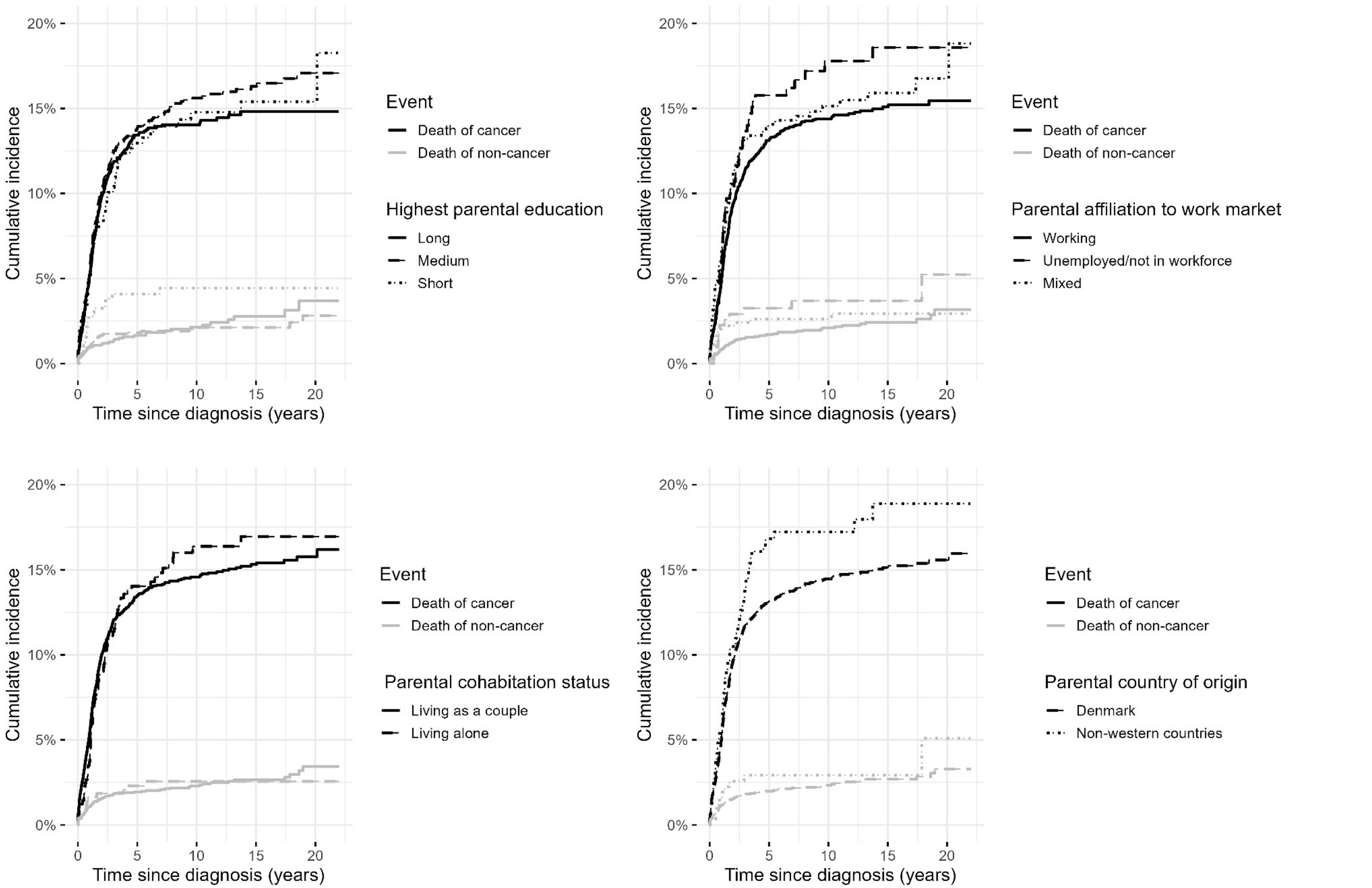

Table 1: Categorization of childhood cancer types based on the ICCC 3rd version diagnostic groups

**Categories**	**Diagnostic groups (ICCC 3rd version)**
Hematological malignancies	1. Leukemias, myeloproliferative and myelodysplastic disease
	2. Lymphomas and reticuloendothelial neoplasms
CNS tumors	3. CNS and miscellaneous intracranial and intraspinal neoplasms
Non-CNS solid tumors	4. Neuroblastoma and other peripheral nervous cell tumors
	5. Retinoblastoma
	6. Renal tumors
	7. Hepatic tumors
	8. Malignant bone tumors
	9. Soft tissue and other extraosseous sarcomas
	10. Germ cell tumors, trophoblastic tumors and neoplasms of gonads
	11. Other malignant epithelial neoplasms and malignant melanomas
	12. Other and unspecified malignant neoplasms

Table 2: Classification of cause of death

Cause of death	ICD-10	ICD-8
**Death of cancer**	C00-97	All ICD-8 codes except 958
**Death of other causes**	Non-cancer natural deaths: A,B,D,E,G,I,J,L,M,N,P,Q,R<br>Suicide: X64-X70<br>Other non-natural deaths: V03, V23, V43, V479, W18, W22, Y658	Other non-natural deaths: 7958

Table 3: Overall 5-year survival in children with childhood cancer by age groups

	0 – 4 years at diagnosis[a]						5 – 10 years at diagnosis[a]						11 – 15 years at diagnosis[a]					
			Unadjusted		Adjusted				Unadjusted		Adjusted				Unadjusted		Adjusted	
	Person years	Events (N)	HR	95% CI[b]	HR[c,d]	95% CI[b]	Person years	Events (N)	HR	95% CI[b]	HR[c,d]	95% CI[b]	Person years	Events (N)	HR	95% CI[b]	HR[c,d]	95% CI[b]
**Highest parental education**																		
Long	3,091	101	1.00	Ref.	1.00	Ref.	1,768	68	1.00	Ref.	1.00	Ref.	1,444	52	1.00	Ref.	1.00	Ref.
Medium	2,468	90	1.12	0.85, 1.49	1.00	0.75, 1.35	1,725	66	1.02	0.73, 1.43	0.91	0.64, 1.29	1,712	59	0.97	0.67, 1.41	0.91	0.62, 1.32
Short	730	25	1.05	0.68, 1.63	0.90	0.57, 1.44	505	19	1.00	0.60, 1.66	0.83	0.48, 1.43	328	20	1.68	1.01, 2.82	1.44	0.83, 2.51
**Parental affiliation to work market**																		
Working	4,379	139	1.00	Ref.	1.00	Ref.	3,031	110	1.00	Ref.	1.00	Ref.	2,844	100	1.00	Ref.	1.00	Ref.
Unemployed/not in work force	585	26	1.35	0.89, 2.06	1.24	0.79, 1.95	39	21	1.46	0.91, 2.32	1.48	0.89, 2.47	297	14	1.34	0.77, 2.35	1.10	0.60, 2.01
Mixed	1,324	51	1.20	0.87, 1.65	1.17	0.84, 1.62	574	22	1.06	0.67, 1.68	1.07	0.67, 1.71	343	17	1.37	0.82, 2.30	1.25	0.73, 2.13
**Parental cohabitation status**																		
Living as a couple	5,546	192	1.00	Ref.	1.00	Ref.	3,325	125	1.00	Ref.	1.00	Ref.	2,830	103	1.00	Ref.	1.00	Ref.
Living alone	743	24	0.93	0.61, 1.43	0.93	0.60, 1.42	673	28	1.11	0.74, 1.67	1.12	0.74, 1.69	654	28	1.16	0.77, 1.77	1.21	0.79, 1.84
**Parental country of origin**																		
Denmark	5,336	176	1.00	Ref.	1.00	Ref.	3,365	129	1.00	Ref.	1.00	Ref.	3,015	106	1.00	Ref.	1.00	Ref.
Western countries or mixed countries	388	10	0.78	0.41, 1.47	0.90	0.47, 1.70	229	8	0.89	0.44, 1.82	0.97	0.47, 2.00	156	8	1.46	0.71, 2.99	1.52	0.74, 3.13
Non-Western countries	565	30	1.57	1.06, 2.31	1.70	1.13, 2.54	405	16	1.02	0.60, 1.71	0.98	0.57, 1.69	313	17	1.53	0.92, 2.55	1.46	0.85, 2.51

[a]Event of overall survival was defined as death from all causes.

[b]Corresponding 95% confidence intervals.

[c]Highest parental education, parental affiliation to work market and parental cohabitation status were adjusted for year of diagnosis, age of mother at time of diagnosis and parental country of origin.

[d]Parental country of origin was adjusted for year of diagnosis, age of mother at time of diagnosis and highest parental education.

Table 4: Overall 5-year survival in children with childhood cancer by sex

	**Females[a]**						**Males[a]**					
			**Unadjusted**		**Adjusted**				**Unadjusted**		**Adjusted**	
	**Person years**	**Events (N)**	**HR**	**95% CI[b]**	**HR[c,d]**	**95% CI[b]**	**Person years**	**Events (N)**	**HR**	**95% CI[b]**	**HR[c,d]**	**95% CI[b]**
**Highest parental education**												
Long	3,023	114	1.00	Ref.	1.00	Ref.	3,280	107	1.00	Ref.	1.00	Ref.
Medium	2,664	106	1.06	0.82, 1.38	1.01	0.77, 1.32	3,241	109	1.05	0.80, 1.37	0.92	0.70, 1.21
Short	663	28	1.12	0.74, 1.69	1.04	0.67, 1.59	900	36	1.24	0.85, 1.81	1.02	0.68, 1.52
**Parental affiliation to work market**												
Working	4,744	176	1.00	Ref.	1.00	Ref.	5,510	173	1.00	Ref.	1.00	Ref.
Unemployed/not in work force	582	31	1.40	0.95, 2.05	1.31	0.87, 1.97	695	30	1.36	0.92, 2.01	1.23	0.81, 1.89
Mixed	1,025	41	1.07	0.76, 1.51	1.06	0.75, 1.51	1,217	49	1.27	0.93, 1.75	1.22	0.87, 1.70
**Parental cohabitation status**												
Living as a couple	5,457	210	1.00	Ref.	1.00	Ref.	6,244	210	1.00	Ref.	1.00	Ref.
Living alone	893	38	1.11	0.78, 1.56	1.09	0.77, 1.54	1,177	42	1.05	0.76, 1.47	1.09	0.78, 1.52
**Parental country of origin**												
Denmark	5,464	203	1.00	Ref.	1.00	Ref.	6,251	208	1.00	Ref.	1.00	Ref.
Western countries or mixed countries	332	17	1.35	0.82, 2.21	1.44	0.87, 2.37	441	9	0.61	0.31, 1.19	0.69	0.35, 1.35
Non-Western countries	554	28	1.31	0.89, 1.95	1.38	0.92, 2.07	729	35	1.43	1.00, 2.05	1.52	1.04, 2.21

[a]Event of overall survival was defined as death from all causes.

[b]Corresponding 95% confidence intervals.

[c]Highest parental education, parental affiliation to work market and parental cohabitation status were adjusted for year of diagnosis, age of mother at time of diagnosis and parental country of origin.

[d]Parental country of origin was adjusted for year of diagnosis, age of mother at time of diagnosis and highest parental education.

Table 5: Cause-specific death in children with childhood cancer followed up to 22 years from date of diagnosis

	Death of cancer[a]						Death of other causes than cancer[*]			
			Unadjusted		Adjusted		Unadjusted		Adjusted	
	Person years	Events (N)	HR	95% CI[b]	HR[c,d]	95% CI[b]	HR	95% CI[b]	HR[c,d]	95% CI[b]
**Highest parental education**										
Long	13,905	208	1.00	Ref.	1.00	Ref.	1.00	Ref.	1.00	Ref.
Medium	14,764	216	1.10	0.91, 1.33	1.02	0.84, 1.24	0.97	0.59, 1.59	0.94	0.57, 1.57
Short	3,724	54	1.05	0.78, 1.42	0.92	0.66, 1.26	1.97	1.08, 3.59	1.94	1.02, 3.71
**Parental affiliation to work market**										
Working	24,264	343	1.00	Ref.	1.00	Ref.	1.00	Ref.	1.00	Ref.
Unemployed/not in work force	2,844	53	1.24	0.93, 1.66	1.20	0.88, 1.65	1.89	1.01, 3.54	1.92	0.96, 3.84
Mixed	5,285	83	1.11	0.87, 1.41	1.09	0.85, 1.40	1.34	0.75, 2.38	1.36	0.75, 2.47
**Parental cohabitation status**										
Living as a couple	27,827	402	1.00	Ref.	1.00	Ref.	1.00	Ref.	1.00	Ref.
Living alone	4,567	76	1.08	0.84, 1.37	1.13	0.87, 1.45	1.05	0.57, 1.94	1.06	0.56, 2.01
**Parental country of origin**										
Denmark	28,206	396	1.00	Ref.	1.00	Ref.	1.00	Ref.	1.00	Ref.
Western countries or mixed countries	-	-	-	-	-	-	-	-	-	-
Non-Western countries	2,724	54	1.25	0.94, 1.66	1.37	1.02, 1.84	1.42	0.73, 2.77	1.28	0.64, 2.57

[*] Due to few observations in some groups, we have refrained from providing any numbers related to non-cancer deaths.

[a]Death of cancer was estimated with death of non-cancer as competing risk.

[b]Corresponding 95% confidence intervals.

[c]Highest parental education, parental affiliation to work market and parental cohabitation status were adjusted for year of diagnosis, age of mother at time of diagnosis and parental country of origin.

[d]Parental country of origin was adjusted for year of diagnosis, age of mother at time of diagnosis and highest parental education.

STROBE Statement — Checklist of items that should be included in reports of cohort studies

	Item No	Recommendation	Page No
**Title and abstract**	1	(*a*) Indicate the study's design with a commonly used term in the title or the abstract	1
		(*b*) Provide in the abstract an informative and balanced summary of what was done and what was found	2
**Introduction**			
Background/rationale	2	Explain the scientific background and rationale for the investigation being reported	3
Objectives	3	State specific objectives, including any prespecified hypotheses	3-4
**Methods**			
Study design	4	Present key elements of study design early in the paper	3-5
Setting	5	Describe the setting, locations, and relevant dates, including periods of recruitment, exposure, follow-up, and data collection	4-5
Participants	6	(*a*) Give the eligibility criteria, and the sources and methods of selection of participants. Describe methods of follow-up	4-6
		(*b*) For matched studies, give matching criteria and number of exposed and unexposed	
Variables	7	Clearly define all outcomes, exposures, predictors, potential confounders, and effect modifiers. Give diagnostic criteria, if applicable	5-6 and Supplementary Figure 2
Data sources/ measurement	8*	For each variable of interest, give sources of data and details of methods of assessment (measurement). Describe comparability of assessment methods if there is more than one group	4-7
Bias	9	Describe any efforts to address potential sources of bias	6-7
Study size	10	Explain how the study size was arrived at	5 and Supplementary Figure 1
Quantitative variables	11	Explain how quantitative variables were handled in the analyses. If applicable, describe which groupings were chosen and why	4-7
Statistical methods	12	(*a*) Describe all statistical methods, including those used to control for confounding	5-7
		(*b*) Describe any methods used to examine subgroups and interactions	5-7
		(*c*) Explain how missing data were addressed	5-7
		(*d*) If applicable, explain how loss to follow-up was addressed	5-7
		(*e*) Describe any sensitivity analyses	6-7

**Results**			
Participants	13*	(a) Report numbers of individuals at each stage of study—eg numbers potentially eligible, examined for eligibility, confirmed eligible, included in the study, completing follow-up, and analysed	7
		(b) Give reasons for non-participation at each stage	
		(c) Consider use of a flow diagram	Supplementary Figure 1
Descriptive data	14*	(a) Give characteristics of study participants (eg demographic, clinical, social) and information on exposures and potential confounders	Table 1
		(b) Indicate number of participants with missing data for each variable of interest	Table 1
		(c) Summarise follow-up time (eg, average and total amount)	7, Table 2 and Table 3
Outcome data	15*	Report numbers of outcome events or summary measures over time	Table 2 and Table 3

Main results	16	(*a*) Give unadjusted estimates and, if applicable, confounder-adjusted estimates and their precision (eg, 95% confidence interval). Make clear which confounders were adjusted for and why they were included	7-9, Supplementary Figure 2, Table 2, and Table 3
		(*b*) Report category boundaries when continuous variables were categorized	7
		(*c*) If relevant, consider translating estimates of relative risk into absolute risk for a meaningful time period	
Other analyses	17	Report other analyses done—eg analyses of subgroups and interactions, and sensitivity analyses	7-9, Supplementary: Figure 3, Table 3, Table 4, and Table 5
**Discussion**			
Key results	18	Summarise key results with reference to study objectives	9-10
Limitations	19	Discuss limitations of the study, taking into account sources of potential bias or imprecision. Discuss both direction and magnitude of any potential bias	13-14
Interpretation	20	Give a cautious overall interpretation of results considering objectives, limitations, multiplicity of analyses, results from similar studies, and other relevant evidence	9-13
Generalisability	21	Discuss the generalisability (external validity) of the study results	13-14
**Other information**			
Funding	22	Give the source of funding and the role of the funders for the present study and, if applicable, for the original study on which the present article is based	15
